# Mobile phone text message intervention to reduce binge drinking among young adults: study protocol for a randomized controlled trial

**DOI:** 10.1186/1745-6215-14-93

**Published:** 2013-04-03

**Authors:** Brian Suffoletto, Clifton W Callaway, Jeffrey Kristan, Peter Monti, Duncan B Clark

**Affiliations:** 1 Department of Emergency Medicine, University of Pittsburgh, Iroquois Building, Suite 400A 3600 Forbes Avenue, Pittsburgh, PA 15261, USA; 2 Center for Alcohol and Addiction Studies, Brown University, Box G-S121-5, Providence, RI, 02912, USA; 3 Western Psychiatric Institute and Clinic, 3811 O'Hara Street, Pittsburgh, PA, 15213, USA

**Keywords:** Alcohol misuse, Intervention, Randomized controlled trial, Effectiveness

## Abstract

**Background:**

Heavy episodic (binge) drinking is common among young adults and can lead to injury and illness. Young adults who seek care in the Emergency Department (ED) may be disproportionately affected with binge drinking behavior, therefore provide an opportunity to reduce future risk through screening, brief intervention and referral to treatment (SBIRT). Mobile phone text messaging (SMS) is a common form of communication among young adults and has been shown to be effective at providing behavioral support to young adult drinkers after ED discharge. Efficacy of SMS programs to reduce binge drinking remains unknown.

**Methods/Design:**

We will conduct a three parallel arm, randomized trial. A convenience sample of adults aged 18 to 25 years attending three EDs in Pittsburgh, PA and willing to participate in the study will be screened for hazardous alcohol consumption. Participants identified as hazardous drinkers will then be allocated to either 12 weeks of weekly SMS drinking assessments with feedback (SA+F), SMS drinking assessments without feedback (SA), or a control group. Randomization will be via an independent and remote computerized randomization and will be stratified by study site. The SA+F group will be asked to provide pre-weekend drinking intention as well as post-weekend consumption via SMS and will receive feedback messages focused on health consequences of alcohol consumption, personalized normative feedback, protective drinking strategies and goal setting. Follow-up data on alcohol use and injury related to alcohol will be collected through a password-protected website three, six and nine months later. The primary outcome for the study is binge drinking days (≥4 drinks for women; ≥5 drinks for men) during the previous month, and the main secondary outcome is the proportion of participants who report any injury related to alcohol in the prior three months.

**Discussion:**

This study will test the hypothesis that a mobile phone text-messaging program will result in immediate and durable reductions in binge drinking among at-risk young adults. By testing an intervention group to an assessment-only and control group, we will be able to separate the effect of assessment reactivity. By collecting pre-weekend drinking intentions and post-weekend consumption data in the SA+F group, we will be able to better understand mechanism of change.

**Trial registration:**

Clinicaltrials.gov NCT01688245

## Background

Excessive alcohol consumption, primarily in the form of binge drinking (≥4 drinks for women; ≥5 drinks for men, per occasion), is responsible for an average of 80,000 deaths in the United States each year [1]. Binge drinking also is associated with a range of social problems, such as motor vehicle crashes and interpersonal violence [2]. In 2010, the National Survey on Drug Use and Health demonstrated that young adults have the highest prevalence of hazardous alcohol use among all age groups, including 41% that had drank ≥5 drinks on a single occasion in the prior month [3]. Young adults also report higher rates of alcohol-related problems than those who do not binge [4,5]. Despite the high rates of binge drinking and associated harms among young adults, two-thirds do not believe that having five or more drinks per week is risky behavior and most do not seek help for hazardous use [6,7]. For these reasons, hazardous drinking among young adults often goes unrecognized, and overall drinking rates among young adults have remained relatively unchanged since 1990 [8,9].

Each day in the United States, there are over 50,000 emergency department (ED) visits by young adults 18 to 24 years of age [10]. A quarter of young adults use the ED for primary care [11] and up to a half may have hazardous alcohol use patterns [12]. For these reasons, the ED provides an opportunistic setting to identify young adults with hazardous alcohol use and intervene to prevent associated risks [13]. Routine screening, brief intervention and referral to treatment (SBIRT) for hazardous alcohol use, including young adults, is promoted by the American College of Emergency Physicians [14] and mandated in trauma wards by the American College of Surgeons [15]. Despite this recommendation, numerous issues exist as barriers to widespread implementation.

There are several aspects of ED SBIRT that limit enthusiasm for widespread adoption [16]. First, whereas some studies have shown ED-based brief interventions are effective in reducing short-term alcohol consumption [17,18], others have been less effective [19]. Second, there are considerable variation in scale, approach and content of ED brief interventions used in studies [20]. Third, there are concerns about therapeutic drift if interventions become routine practice [21]. Finally, barriers existing at the patient and provider levels, include lack of comfort discussing sensitive topics like alcohol with a care provider [22] and lack of confidence, training and time to perform SBIRT among providers [23,24]. Given these barriers, innovative techniques are needed to improve adoption of SBIRT.

One promising modality that could assist effective delivery of brief interventions for alcohol use, especially among young adults, is mobile communication technology. Ninety five percent of young adults own a mobile phone and 97% of these use text messaging (SMS) either sending or receiving an average of 50 texts per day [25]. SMS has been used to promote health in a wide range of young adult health issues, including diabetes [26], asthma [27], cigarette smoking [28] and risky sex [29]. Our group has shown that SMS is feasible to communicate with young adults about alcohol use after ED care and shows promise in reducing binge drinking episodes in the short-term [30].

The current study objective is to determine whether an SMS program is effective at reducing and maintaining reductions in binge drinking and related harms among at-risk young adults. Here we present the study protocol for a three parallel arm, randomized controlled phase III trial with a control, assessment-only, and intervention arm. We hypothesize that a 12-week SMS behavioral intervention delivered through SMS will result in reduced number of past-30 day binge drinking episodes at three-, six- and nine-months following care in the Emergency Department. If found to be effective, the automated nature and low cost of the intervention would allow widespread adoption. By comparing an intervention group to an assessment-only group, we will be able to separate the effect of assessment reactivity. By collecting pre-weekend drinking intentions and post-weekend consumption data in the intervention group, we will be able to better understand mechanism of change.

## Methods/Design

### Trial design

**Figure 1:**
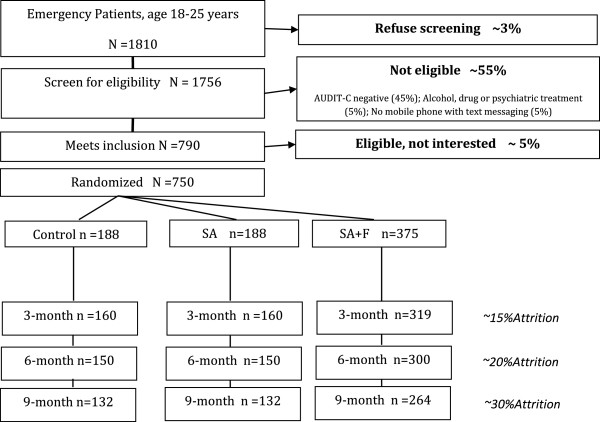
Flowchart for the TRAC Trial.

The Texting to Reduce Alcohol Consumption (TRAC) Trial is a multicenter, three parallel arm, randomized, controlled and double-blind clinical phase III trial with participant recruitment at three EDs in Western Pennsylvania (Pittsburgh). Recruitment started on November 1, 2012. Some 750 ED patients 18 to 25 years of age who report hazardous alcohol use will be randomized to either (1) a 12-week SMS program, where they will be asked to conduct weekly dialog regarding their drinking intentions, consumption and goal setting, or (2) a 12-week assessment-only program, where they will be asked to provide weekly drinking consumption reports without receiving feedback or (3) a control group, who will not receive any SMS drinking assessments. The detailed study design is provided below according to the revised CONSORT statement [31] and flowchart is illustrated in Figure 1. The study is approved by the University of Pittsburgh Institutional Review Board and is registered at http://clinicaltrials.gov (NCT01688245). All participants are required to give informed written consent.

### Setting

Recruitment is occurring within three EDs at the University of Pittsburgh Health Care System, including two Level I trauma centers and one Level II trauma center. Patient characteristics from the fiscal year 2011 include an average of 11,349 patients aged 18 to 25 years of age, among whom 64% were female, 72% Caucasian, and 24% African American. Hispanic ethnicity was estimated as 2%.

### Recruitment

A Research Associate (RA) will be stationed in the EDs seven days a week, with 60% evening shifts (4 pm to 12 am) and 40% day shifts (8 am to 4 pm). The RA will identify potential participants 18 to 25 years of age and not critically ill using an electronic triage board. The RA will then obtain permission from the ED clinician to approach a potential participant in their treatment room. If a potential participant is still intoxicated, the RA will wait until the clinician declares that the subject is no longer intoxicated. The RA then explains the rationale for the study, stating that we are conducting a study about alcohol use among young adults. The researcher will encourage potential participants to spend as much time as they want asking questions about the study and considering whether they wish to take part or not. If the patient is interested in participating, informed consent is obtained and a nine-item self-administered screening instrument is completed using a tablet computer to minimize social desirability bias in reporting sensitive information.

### Participants

Inclusion criteria for participation in the TRAC trial comprise the following: age 18 to 25 years, English-speaking, hazardous drinking behavior (Alcohol Use Disorder Identification Test for Consumption: AUDIT-C scores of >3 for women or >4 for men) [32] and last month binge episode. Exclusion criteria include the following: past treatment for drug or alcohol disorder, current treatment for psychiatric disorder (including depression). Participants are also excluded if they do not own a cell phone with text messaging. Young adults with hazardous drinking who are not eligible or decline study participation are provided the same referral and educational materials as study participants, but they will not participate in any further assessments or monitoring. These young adults are directed to contact their primary care physician or a local community resource about reducing their alcohol consumption. Eligible and interested participants sign and date the Informed Consent Form prior to any further baseline clinical and demographic data, randomization and treatment allocation. We collect multiple points of contact from each participant, including at least one ‘locator’ if we cannot find the participant for follow-up.

### Randomization

Randomization assignments were generated in blocks of eight for each site by the study statistician and allocated electronically. Randomization sequences are stratified by site in a 2 intervention:1 assessment:1 control ratio to allow for more observations to be available for analyses of mechanisms of change in the intervention group. Participants are blinded to treatment allocation to minimize expectation bias.

### SMS-drinking assessments with feedback (SA+F) intervention

The SA+F intervention is based on that used in a previous trial [30], and targets the following key determinants of binge drinking behavior: intention to binge drink, knowledge of health risks associated with binge drinking, norms of drinking in their age group, skills to reduce binge drinking and action planning to avoid a binge episode. The behavior techniques aimed at modifying these behavioral determinants utilizes elements of the Health Belief Model [33], the Information Motivation Behavior model [34] and the Theory of Reasoned Action and Planned Behavior [35] including: information linking drinking behavior to consequences and health, intention formation, barrier identification, general encouragement, goal setting, self-monitoring, positive feedback on performance, and rolling with resistance. The dialog and messages were developed by a multidisciplinary team of emergency physicians (BS, CC) alcohol treatment specialists (DC, PM), partially adapted from written educational material from the National Institute on Alcohol Abuse and Alcoholism (NIAAA’s) ‘Rethinking Drinking’ [36] and attempted to reflect the style of language used in motivational interviewing [37]. Messages were pilot tested in our target population and were qualitatively assessed by a separate population of 400 young adult drinkers (unpublished data).

Upon entering their phone number into our system, intervention participants receive a series of welcome text messages describing the program: ‘Welcome to TRAC Study. Our job is to help you reduce your drinking to improve your health. The National Institute of Health considers drinking more than four drinks for men or three for women to be a health risk. For completion of each day’s assessments, we will add $1 to your account, which you can access after three-, six-, and nine-month follow-ups.’ Each Thursday at 4 pm, intervention participants receive a sequence of text messages to assess their drinking plan: ‘Hey, it’s the TRAC Research Team checking in. Do you plan on drinking this weekend?’ If they reply ‘YES’ or equivalent, they receive: ‘Do you think that you are likely to have more than (x) ((three) for women (four) for men) drinks on any one occasion?’ If they reply ‘YES’ or equivalent, they receive: ‘Would you be willing to set a goal to drink less than (y) ((four) for women (five) for men) drinks when you are drinking?’ Depending on their response, they receive either positive reinforcement with provision of protective drinking strategies or encouragement to them to reflect on their decision.

On Sunday at 12 pm, intervention participants receive a sequence of text messages to assess their alcohol consumption: ‘Hey, it’s the TRAC Research Team checking in. Between Thursday and today, what is the most number of drinks you had on any drinking occasion?’ If they report at least one drink, they receive: ‘About over how many hours did you have this many drinks?’ Depending on their response, they receive a text message feedback focused on the health consequences of their alcohol consumption and normative feedback. At the completion of the feedback, they then receive: ‘Thanks for completing this day’s assessment. We have added $1 to your account. Only (x) weeks to go.’ where (x) is the number of weeks remaining.

### SMS drinking assessments without feedback (SA)

Individuals in the SA group will not receive any pre-weekend assessments but will receive identical post-weekend SMS drinking assessments to the intervention group at 12 pm on Sunday for 12 weeks. Upon receiving their responses, the computer will send the following text message: ‘Your response has been received and is appreciated.’ This group is critical to separate the effect of the intervention from that associated with assessment reactivity [38].

### Control

Individuals in the control group will not receive any SMS drinking assessments.

### Remuneration

Participants are given the following material incentives to remain in the study: they are reimbursed $10 for completing the baseline assessments, $20.00 for completing the three-month web-based assessment, $30.00 for completing the six-month web-based assessments and $40.00 for completing the nine-month web-based assessments. For the SA+F and SA group, the cost of text messaging is covered in the flat fees for reimbursement for participation. The SA+F group has the opportunity to receive an additional $1.00 per completed Thursday and Sunday SMS dialog, for a maximum of $24.00 additional payment. We believe that the additional payment to the SA+F group would incentivize adherence to SMS dialog, which could have the intended effect of optimizing behavior change through improved self-monitoring.

## Measures

### Baseline measures

Basic demographic data on age, gender, race, ethnicity and current enrollment in school will be collected from all patients who agree to initial screening. These will be used to describe the basic differences between those who do and do not report hazardous drinking. Alcohol consumption will be assessed using the AUDIT-C, which consists of three questions: 1. How often do you have a drink containing alcohol? 2. How many drinks containing alcohol do you have on a typical day when you are drinking? and 3. How often do you have six or more drinks on one occasion? The AUDIT-C parallels or exceeds comparable instruments used in clinical and research settings for the detection of hazardous drinking [12] and a cut point of >4 (or >3 for females) produces a sensitivity of 0.76 to 0.99, and specificity of 0.65 to 0.98 [39]. Additionally, we will ask: ‘Have you had any day over the last month where you drank more than three (four for men) drinks containing alcohol?’ to differentiate those with current binge drinking from those without. In our pilot study [30], we found that 18% of young adults (despite meeting AUDIT-C criteria for hazardous drinking) did not have any episodes of binge drinking during the month prior to enrollment, potentially masking treatment effects at follow-up. We will also ask the ED physician: ‘In your opinion, was this patient visit to the ED attributable to alcohol?’ and ‘Did the patient present to the ED intoxicated?’ which will be examined as potential moderators of treatment effectiveness.

For those patients who are eligible and enroll in the TRAC trial, we will collect further demographics including co-habitation (‘Who do you live with?’), marital status, and current employment. Alcohol consumption will be measured using the Timeline Followback (TLFB) procedure [40]. Using a calendar, participants provide retrospective estimates of their daily drinking over the 30 days prior to the interview date. Memory aids will be used to enhance recall (for example, visual calendars with key dates and holidays serve as anchors for reporting drinking; a visual chart of standard drink sizes reduces variability in quantity). Alcohol-related injuries are measured using the Injury Behavior Checklist (IBC) [41]. The IBC asks participants about how often each of the 17 injuries had occurred in the past three months. If a participant affirms that an injury had happened, subsequent questions about the use of alcohol prior to injury occurrence and whether they were treated by a doctor are asked. Drug use in the past three months is measured using the National Institute on Drug Abuse (NIDA)-modified Alcohol, Smoking and Substance Involvement Screening Test (ASSIST). The ASSIST was developed by the World Health Organization and developed principally for use in primary health care settings [42]. Depression and anxiety are measured using a two-item depression scale and a two-item anxiety scale, that is, the Patient Health Questionnaire for Depression and Anxiety (PHQ-4) [43]. Drinking norms are measured through a single question: ‘How much do you drink compared to others your age?’ where responses can range from ‘much less’ to ‘much more’, anchored by option ‘the same’. Behavioral control is measured through the question: ‘How sure are you that you could resist “drinking to get drunk” when out with friends?’ where responses can range from ‘not sure at all’ to ‘completely sure’.

### Follow-up measures

Follow-up data will be obtained through a password-protected website at three-, six- and nine-months after ED discharge. At each time point, alcohol consumption data in the last 30 days will be collected using the TLFB procedure. Alcohol-related injuries are measured using the IBC, drug use measured using the ASSIST, depression and anxiety using the PHQ-4, and drinking norms and behavioral control through identical questions to baseline. At nine-month follow-up, all participants will complete a structured diagnostic assessment for determining Diagnostic and Statistical Manual of Mental Disorders, 4th Edition (DSM-IV) diagnoses for alcohol abuse and dependence. Individuals who meet alcohol abuse or dependence criteria will also receive alcohol treatment referrals.

### Outcomes

The primary outcome is the change in mean number of binge drinking days during the previous 30 days from baseline to follow-up using the TLFB. The main secondary outcome is the change in the proportion of participants with at least one alcohol-related injury (IBC) from baseline to follow-up. Other secondary outcomes include the mean number of drinks consumed per drinking day. We will examine the potential cognitive and motivational processes that mediate the influence of our intervention on alcohol consumption, specifically pre-weekend drinking intentions and goal-setting rates over the course of the 12-week SMS dialog. We will explore whether ED presentation related to alcohol and co-occurring drug use at baseline moderates the efficacy of our intervention.

### Sample size

For power consideration, we focus on detecting the difference between intervention and control groups in binge drinking days, since it is the primary comparison of interest. Using information from our pilot study [30], if we assume the number of binge drinking days in the intervention group will decrease by a mean of 3.4 (SD 5.4) from baseline to three-month follow-up compared to a decrease of 1.1 (SD 4.1) in the control group, using a sample size ratio of 2:1, we will need 96 participants in the intervention and 48 in the control group to have 80% power to show a difference at significant level = 0.05 based on two-sided two-sample *t* test. Including 48 participants in the assessment group to follow our planned sample ratio 2:1:1, we will need 192 subjects in total. Allowing for a 25% attrition rate, we will actually need to enroll 256 participants in order to achieve a target sample size of 192. If we consider binge drinking days at 9 months, assuming the change will decrease by 35% at 9 months, we need 750 subjects, allowing for 30% attrition. In our pilot, 49% of those screened were hazardous drinkers who scored positive on the AUDIT-C, and 87% of those actually enrolled. So in order to enroll 750 we will thus approach 1760 patients for study participation.

### Statistical analysis

Initially, we will describe the population and all treatment group data graphically and numerically in order to confirm normality for continuous measures, identify expected ranges, detect outliers, and assess data variability. We will specifically examine comparability of treatment groups at baseline, patterns of participant dropout and missing data. In the event that treatment groups differ on baseline sociodemographic characteristics, alcohol-related, and risk-related variables, we will run analyses both with and without these variables as covariates to determine whether baseline differences may account for differences in outcomes. Attrition will be evaluated for systematic differences between patients who complete the research and those who drop out. In this way, we can determine the nature of the potential bias introduced by attrition. Dependent variables will be examined to determine which distributional models are most appropriate. We will use generalized estimating equation analyses for the major study aims and, depending on the nature of the variable, the analyses will use either a normal distribution or negative binomial model for count data (for example, number of binge drinking days). Our models will account for clustering within each subject and each site. If any covariates are identified as necessary during preliminary analyses, these will also be included as covariates. After testing the main effects of condition, we will test interactions between intervention and time to inform us whether differences in alcohol use and consequences outcomes associated with intervention become less or more pronounced over nine months. Following ‘intention-to-treat’ principles, analyses will be conducted on all who were randomized to a condition regardless of whether they actually completed the intervention sessions.

If the intervention does result in significantly lower rates of alcohol use and/or alcohol-related adverse consequences, analyses will be conducted separately to determine whether these effects are mediated by pre-weekend drinking intention and SMS goal-setting patterns. For a variable to be a mediator (a) intervention condition must be significantly related to the intermediate variable; (b) the intermediate variable must be significantly related to alcohol outcome; (c) intervention condition must be significantly related to alcohol outcome; and (d) after controlling for the intermediate variable, the relationship between intervention condition and outcome is significantly reduced. Product of coefficients methods will be used to determine the size and significance of the reduction in the effect of intervention due to inclusion of the mediators. We will also conduct exploratory analyses of potential moderators of the intervention. We will be able to explore whether baseline ED presentation attributable to alcohol and drug use is an important moderator of change in drinking behaviors in addition to the demographic moderators. These will be completed with another set of multivariable regression models. All analyses will be performed with two-sided *P* values considered significant when below 0.05.

## Discussion

The TRAC study is the first large-scale randomized trial to examine the effectiveness of a text message program for initiating and maintaining reductions in binge drinking among at-risk young adults. If found to be effective, our intervention could be easily scaled-up as a stand-alone program to provide widespread support with minimal disruption to normal clinical practice. Additionally, it could easily be included as a piece in a multi-component intervention, such as a “booster” to traditional in-person counseling interventions. By incorporating longitudinal assessments of drinking behavior in a patient’s natural environment through SMS, stepped-care models could be built where individuals who continue to report hazardous use after program exposure could then be referred directly to a help line or counselor.

There are several potential strengths of our research design worth mentioning. In addition to being large enough to demonstrate reductions in alcohol consumption immediately following intervention (three-month follow-up) as well six months later (nine-month follow-up), we have sufficient power to examine mediators of change associated with this intervention. Inclusion of an assessment-only group minimizes the potential for assessment effects to bias estimates of intervention efficacy. While all participants complete informed consent, where we outline the various treatments they may be subjected to, we do not specifically tell them which one. We believe that this may reduce expectation biases. All baseline measures and outcomes are reported to an automated computer system, potentially increase disclosure of potentially sensitive or embarrassing information, thus reducing reporting biases. By collecting pre-weekend drinking intentions/plans, goal-setting/plan changes, and post-weekend consumption in the intervention group, we will be able to examine “micro processes” or mechanisms of change not previously available in brief intervention studies. Finally, we have made efforts to design our mobile intervention both from a theory-driven approach to behavior change [44] and a user-centered approach [45], which will serve the dual purposes of increasing potential effectiveness as well as reducing intervention attrition.

The major methodological challenge in this study relates to retention of participants. ED-based brief intervention studies may have lost to follow-up rates as high as 40% [19]. Accordingly, we have adopted a method of obtaining multiple points of contact from participants, conducting follow-up through a web-based platform, and analytic methods for estimating the degree of any attrition bias and potentially adjusting for it. If we are able to meet these challenges, we will generate data about a mobile behavioral program that has the potential to improve the health of young adults outside of traditional care settings.

## Trial status

Active recruitment

## Competing interest

All authors have no competing interests.

## Authors’ contributions

BS, JK, CC and DC conceived the study and designed the trial. PM provided critical design considerations for the intervention. JK is supervising all recruitment and enrollment. All authors contributed substantially to the manuscript’s revision. BS takes responsibility for the paper as a whole. All authors read and approved the final manuscript.
